# Vitamin D levels and left ventricular function in beta-thalassemia major with iron overload

**DOI:** 10.1007/s00431-023-04830-7

**Published:** 2023-02-10

**Authors:** Mrudula Pala, Kamalakshi G. Bhat, Sharath Manya, Nitin Joseph, Sindhu Harish

**Affiliations:** 1 Department of Pediatrics, Kasturba Medical College, Mangalore, Manipal Academy of Higher Education, Manipal, India; 2 Department of Community Medicine, Kasturba Medical College, Mangalore, Manipal Academy of Higher Education, Manipal, India; 3 Department of Biochemistry, Kasturba Medical College, Mangalore, Manipal Academy of Higher Education, Manipal, India

**Keywords:** Thalassemia, Iron overload, Vitamin D, Fractional shortening, Ejection fraction

## Abstract

Heart disease is the primary cause of death in patients with beta-thalassemia major. The study aimed to determine the association between vitamin D and left ventricular function in patients with beta-thalassemia major with iron overload. A cross-sectional hospital-based study was conducted, where the vitamin D and ferritin levels of children living with beta-thalassemia major were measured, and left ventricular function was assessed utilizing ejection fraction (EF) and fractional shortening (FS) using 2D echocardiography. The mean serum ferritin was 4622 ± 2289 ng/ml, and the mean serum vitamin D levels were 22 ± 7.7 ng/ml. The mean values of EF were 62.30 ± 6.9%, and FS was 31.21 ± 4.8%. Statistically significant negative correlation (*r *= −0.447, *p *< 0.001) was found between vitamin D and serum ferritin values, and a significant positive association was found between vitamin D levels concerning EF and FS with a p-value of 0.034 and 0.014, respectively.

*Conclusion*: It was observed  that increasing ferritin was associated with lower vitamin D levels which in turn influenced fractional shortening /cardiac function in these patients.

**Table:** 

**What is Known:** *• Patients with Beta Thalassemia major on long term transfusion are prone to develop heart disease / cardiac failure due to chronic iron overload.*	
**What is New:** *• Patients with beta thalassemia major on long term term transfusions with iron overload who are vitamin D deficient are more prone to the cardiac complications which inturn can be prevented by vitamin D supplementation.*

**What is Known:**

*• Patients with Beta Thalassemia major on long term transfusion are prone to develop heart disease / cardiac failure due to chronic iron overload.*

**What is New:**

*• Patients with beta thalassemia major on long term term transfusions with iron overload who are vitamin D deficient are more prone to the cardiac complications which inturn can be prevented by vitamin D supplementation.*

## Introduction

β-Thalassemia major is an inherited hemolytic anemia characterized by impaired production of beta-globin chains. It leads to ineffective erythropoiesis requiring frequent blood transfusions leading to iron overload. Iron overload affects several endocrine organs, skin, and kidneys, leading to vitamin D deficiency. A recent review article reveals the prevalence of vitamin D deficiency varies from 24.8 to 80.6%. All major thalassemia patients have a vitamin D deficiency irrespective of age, gender, sex, race, and geographical location [1]. The prevalence of vitamin D deficiency in an average population of the same age group is significantly less compared to thalassemia patients [2, 3].

Vitamin D deficiency stimulates trans-membrane calcium transport through the left ventricular dependent calcium channel (LVDCC), leading to the transport of non-transferrin-bound iron (NTBI) into the myocardium leading to cardiac iron overload [4]. This increases myocyte oxidative stress and disrupts sodium, potassium, and calcium ion channels, causing depolarization and repolarization disturbances, arrhythmias, and systolic and diastolic disturbances.

With this background, we aimed to study the level of vitamin D and the association of vitamin D with left ventricular function in children and adolescents with beta-thalassemia major and iron overload. It would enable early identification of cardiac dysfunction, which could be treated with oral vitamin D supplements.

## Materials and methods

A hospital-based cross-sectional study was conducted at a tertiary care teaching hospital in coastal South India from May 2017 to January 2019.

The study included patients aged 5 to 18 years with beta-thalassemia major with iron overload as indicated by serum ferritin of > 2500 ng/ml (moderate iron overload) in the previous three months [5]. Patients with hemoglobin levels < 8 gm/dl, those with heart disease during echocardiography and preexisting heart diseases, and children on vitamin D supplementation in the last 6 weeks were excluded.

Taking 80% of the prevalence of vitamin D deficiency in thalassemia in India, the sample size was calculated to be 70 [6]. Institutional Ethics Committee approval was obtained before starting the study.

The study procedure was explained to all the participants and their parents. A detailed semi-structured proforma with socio-demographic profile, age of onset of disease, details of transfusions from the time of diagnosis, chelation therapy, growth and development, examination, vitamin D intake, and 2D echocardiographic findings were filled as per standard protocol. A written informed consent/assent was taken from the patients and their parents after explaining the purpose of the study in their local language.

A 2-ml plain blood sample was collected from each subject under aseptic conditions before the transfusion to estimate vitamin D levels by ELISA kit. The serum was extracted and stored in plain vacutainer tubes at − 20 °C at the institutional central research laboratory. Vitamin D levels were measured using a CALBIOTECH kit based on ELISA technology. Estimation of vitamin D was done, and the study subjects were subdivided into vitamin D deficient, insufficient, and sufficient groups based on USIOM Classification and were analyzed [7].

The USIOM classification of vitamin D is depicted in Table 1.

**Table 1:** US IOM classification of vitamin D levels

**Units**	**Vitamin D status**
≤ 5	Severe deficiency
≤ 15	Deficiency
15–20	Insufficiency
20–50	Sufficiency
≥ 50	Intoxification

### Echocardiography

Left ventricular function was assessed utilizing ejection fraction and fractional shortening using 2D echocardiography [8]. 2D echocardiography was performed by a cardiologist using the GE Health Care LOGIQ5 PRO model machine. The probe was a 7S (3–8 MHz) probe.

#### Fractional shortening (FS) is the fraction of any diastolic dimension lost in systole

It was measured by using the formula FS = LVEDD − LVESD/LVEDD, where LVEDD is the left ventricular end-diastolic dimension and LVESD is the left ventricular end-systolic dimension.

The normal range was 26–45% [9].

#### Ejection fraction (EF) refers to the amount or percentage of blood pumped out of the ventricles with each contraction

It was measured by using the formula EF = stroke volume/end-diastolic volume < 54% − below normal ≥ 54% − normal ejection fraction [9].

Data entry and analysis were done using Statistical Package for Social Sciences (SPSS) version 17.0. The association of serum ferritin and vitamin D levels was crosstabled with ejection fraction and fractional shortening. The association between categorical variables was done using the chi-square test and Fisher’s exact test. Correlation between continuous variables was done using Karl Pearson’s coefficient of correlation. *p*-value < 0.05 was considered significant and *p* < 0.01 was highly significant.

## Results

The study included 70 subjects, and the mean age of the study population was 10.0 ± 3.56 years (range of 5 to 18 years), in which patients belonging to the first decade were 35 (50%) and those belonging to the second decade were 35 (50%) as depicted in Table 2.

**Table 2:** Association between the age of the thalassemia major patients with their vitamin D levels (total number of participants = 70)

**Age group (years)**	**Vitamin D levels (ng/ml)**		**Total**
	Insufficient/deficient (%)	Sufficient (%)	
**≤ 7**	5 (27.8)	13 (72.2)	18
**7–11**	10 (41.7)	14 (58.3)	24
**> 11**	15 (53.6)	13 (46.4)	28
**Total**	30	40	70

*X*^2^ = 3.0, *p* = 0.223

There were 33 (47.1%) girls and 37 (52.9%) boys. 55.7% of the patients were diagnosed to have thalassemia major before the age of 1 year. 41.1% were diagnosed between 1 and 5 years of age. The mean age at diagnosis was 1.4 years.

The mean vitamin D levels were 22 ± 7.7 ng/ml. As per the US IOM classification, out of 70, 9 (12.9%) were vitamin D deficient, 22 (31.4%) were vitamin D insufficient, and 39 (55.7%) were vitamin D sufficient.

In the study population, 49 patients had ferritin ranging from 2500 to 5000 ng/ml, and 21 had ferritin above 5000 ng/ml. The mean serum ferritin values were 4622 + 2289 ng/ml. The association between serum ferritin values and vitamin D levels is shown in Table 3.

**Table 3:** Vitamin D levels in relation to ferritin values (total number of participants = 70)

**Vitamin D status**	**Ferritin 2501–5000 ng/ml**	**Ferritin > 5001 ng/ml**	**Total**
**Deficient (< 15 ng/ml)**	4 (44.4%)	5 (55.6%)	9
**Insufficient (15–20 ng/ml)**	12 (54.5%)	10 (45.5%)	22
**Sufficient (> 20 ng/Ml)**	33 (84.6%)	6 (15.4%)	39
**Total**	49	21	70

*X*^2^ = 9.268, *p* = 0.01

**Fig. 1:**
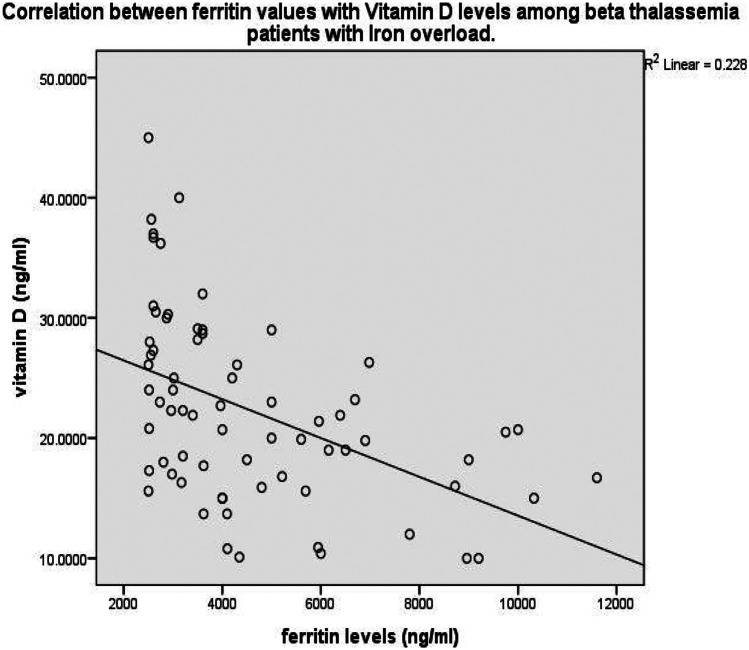
Scatter diagram depicting the correlation between ferritin and vitamin D levels

Statistical analysis using Karl Pearson’s correlation test showed a significant negative correlation between vitamin D and serum ferritin (*p* < 0.001, *R* = − 0.477), indicating that vitamin D level decreases as iron overload increases. The correlation is depicted in the scatter diagram in Fig. 1.

Left ventricular function was assessed utilizing ejection fraction (EF). The mean value was 62.3 ± 6.9%, ranging from a minimum of 43% to a maximum of 76%. In this study, 10 participants had an ejection fraction below normal (< 54%) and 60 participants had an ejection fraction that was expected (> 55%). The association between vitamin D ejection fraction was analyzed using Fisher’s exact test. There was a significant association between vitamin D and EF (*p* = 0.018) (Table 4).

**Table 4:** Association between serum vitamin D levels and left ventricular function in terms of ejection fraction (EF) and fractional shortening (FS) (total number of participants = 70)

**Vitamin D (ng/ml)**	**EF (< 54%)**	**EF (≥ 54%)**	**Total**	**FS (< 25%)**	**FS (≥ 25%)**	**Total**
**≤ 20**	8 (25.8%)	23 (74.2%)	31	5 (16.1%)	26 (83.9%)	31
**> 20**	2 (5.1%)	37 (94.9%)	39	0 (0%)	39 (100%)	39
**Total**	10	60	70	5	65	70

*p* = 0.018 (EF and vit. D levels)

*p* = 0.014 (FS and vit. D levels)

Another component of left ventricular function, fractional shortening (FS) was also measured by 2D echocardiography. The mean value of FS was found to be 31.21 ± 4.805, ranging from 22 to 41%. Fractional shortening was considered normal between 25 and 40% and abnormal when less than 25%. In this study, out of 70 patients, 5 had low fractional shortening less than 25% and 65 were within the normal range. The association between vitamin D and FS was analyzed using the Fisher exact test where a positive association was found between vitamin D and FS (Table 4).

The role of vitamin D and ferritin was analyzed with fractional shortening using binary logistic regression analysis. It was found that vitamin D levels were influencing fractional shortening after adjusting for the confounding effects of ferritin levels (Adjusted odd’s ratio = 4.967, 95% confidence intervals, 1.299–18.998, *p* = 0.019), indicating iron overload in thalassemia major children will cause cardiac dysfunction because of low vitamin D levels in them.

## Discussion

It is well-known that repeated blood transfusions in beta-thalassemia major cause iron overload in vital organs like endocrine organs, heart, liver, and lungs leading to delayed puberty, defective vitamin D synthesis, cardiac dysfunction, pulmonary dysfunction, hypertension, and restrictive lung disease.

Cardiomyopathy due to iron overload is the leading cause of death in chronic transfusion therapy patients [10]. Echocardiographic evidence of ventricular diastolic dysfunction can be detected earlier before systolic dysfunction occurs, using tissue Doppler signals [6, 11]. The present study was designed with this background to assess the association between vitamin D and left ventricular function in thalassemia major with iron overload. The subjects were above 5 years old and with more than 2500 ng/ml serum ferritin. The mean age of the study population was 10.24 + 3.56 years, which is in concordance with the other Indian studies [12, 13]. It also corresponds to another study on thalassemia majors from our institution, where the mean age group is 12.27 ± 4.18 years. Other studies from various populations had a higher mean age [12, 14].

In this study, the mean ferritin level was 4622 ± 2289.87 ng/ml, which is well over 2500 ng/ml, which is associated with an increased risk of cardiac dysfunction. It has been previously reported by Anderson et al. that ferritin levels > 2500 mg/l increased the risk of cardiac dysfunction [15]. After the introduction of chelating agents, the occurrence of heart failure shifted to the second decade. The mean age of our subjects is 10.2 ± 3.56 years; therefore, left ventricular ejection fraction (LVEF) and fractional shortening (FS) were still within normal limits but would begin to decrease as the adolescents would get older.

Vitamin D suppresses tumor necrosis factor α (TNF-α) release and increases interleukin-10 (IL-10) synthesis. Raised inflammatory cytokines like TNF-α and deficient IL-10 lead to severe atherosclerosis. Schleithoff et al. found that compared to placebo, parathyroid hormone levels (PTH) were significantly lower in congestive heart failure (CHF) patients with vitamin D supplementation [16]. IL-10, an anti-inflammatory cytokine, was increased after vitamin D supplementation, and pro-inflammatory cytokine TNF-α remained static in the vitamin D supplementation group. It was raised in the placebo group [16].

In this study, 31 (44.3%) children with thalassemia major had vitamin D deficiency (< 20 ng/mL). These results were consistent with a study by Fadhillah et al. that stated there was vitamin D deficiency in 85.5% of thalassemia children in Hasan Sadikin Hospital [17]. Wood et al. also found vitamin D deficiency occurred in 55.4% of patients with thalassemia [18]. Dipankar Hazarika et al. showed 52% vitamin D deficiency in thalassemia patients [19]. Dhale et al. showed that 77.7% of thalassemia patients had vitamin D levels lower than required [20]. Vitamin D deficiency was present in 82% of the thalassemia patients in contrast to 47% of the standard controls in a study conducted by Akhouri et al. in 2017 [2]. 25-OH-vitamin D deficiency was observed in 98% of the study group of thalassemia patients and 68% in the control group in the Agrawal et al. study in 2016 [3]. Ahmed in 2019 compared vitamin D levels in thalassemia patients (13.12 ± 2.90 ng/ml) with standard values of vitamin D (24.2 ± 4.1 ng/ml) in India among the pediatric population [21]. Iron deposition in the liver and skin of patients with thalassemia major disrupts hydroxylation and synthesis of vitamin D, so most of the patients with thalassemia major have vitamin D deficiency.

No significant correlation was found between age and vitamin D in our study. These results concord with the earlier study by Ambarwati et al. in Indonesia [22]. There is a statistically significant negative correlation between vitamin D levels and serum ferritin. These results were consistent with the findings of Wood et al., who had observed a negative correlation between vitamin D and ferritin levels among thalassemia children [18].

In our study, 2D echocardiography is used to estimate the function of left ventricular function. In a study by Koohi et al., diastolic dysfunction (34%) was the most common echocardiography disorder, while the systolic dysfunction prevalence estimated was about 9% [23]. Davis et al. found that left ventricular diastolic dysfunction develops early, but the systolic dysfunction determines the outcome in these patients, and most die of systolic dysfunction [24].

The results of this study showed a significant positive association between levels of vitamin D and left ventricular function as represented by EF (*p* = 0.018) and FS (*p* = 0.014). This result was consistent with a previous study by Wood that states a correlation between vitamin D levels and LVEF [25]. Low vitamin D levels increase parathyroid hormone production, increasing heart rate. It also disturbs the contraction of cardiomyocytes and increases natriuretic peptide secretion, which may lead to cardiac hypertrophy [26, 27]. Therapy with an active vitamin D analog reduces left atrial hypertrophy and attenuates the rise of BNP [28].

A longitudinal prospective analysis is required to observe the improvement of cardiac function after vitamin D supplementation and chelation in beta-thalassemia children with iron overload. Although this study identified significant associations between vitamin D levels and cardiac status, a longitudinal follow-up was not done. Further studies are required to determine the adequate dose of vitamin D to prevent overload and improve cardiac function. It would help establish standard guidelines for vitamin D supplementation in all beta-thalassemia children.

## Conclusion

To conclude, a significant negative correlation was found between iron overload (ferritin) and vitamin D levels, understanding that increasing ferritin was associated with lower vitamin D levels.

Vitamin D levels influenced fractional shortening after adjusting for the confounding effects of ferritin levels. Hence, vitamin D supplementation needs to be considered for all thalassemia patients particularly those with vitamin D deficiency which might help improve their cardiac function and reduce morbidity and mortality.

## Data Availability

Data is available with the authors.
